# Breast cancer pathogenesis is linked to the intra-tumoral estrogen sulfotransferase (hSULT1E1) expressions regulated by cellular redox dependent Nrf-2/NF*κ*β interplay

**DOI:** 10.1186/s12935-020-1153-y

**Published:** 2020-03-04

**Authors:** Aarifa Nazmeen, Guangping Chen, Tamal Kanti Ghosh, Smarajit Maiti

**Affiliations:** 1 Dept. of Biochemistry, Cell & Molecular Therapeutics Lab, Oriental Institute of Science & Technology, Midnapore, 721101 India; 2 Venture I OSU Laboratory, Oklahoma Technology & Research Park, 1110 S. Innovation Way, Stillwater, OK 74074 USA; 3 Special Secretary, Higher Medical Education, Health and Family Welfare Dept, Govt. of West Bengal, Salt Lake, Calcutta, India; 4 Department of Biochemistry and Biotechnology, Cell & Molecular Therapeutics Lab, OIST, Midnapore, 721102 India

**Keywords:** Breast cancer, SULT1E1 redox regulation, Estrogen, Nrf2 and NFκβ, Xenograft

## Abstract

**Background:**

Estrogen sulfotransferase catalyzes conjugation of sulfuryl-group to estradiol/estrone and regulates E2 availability/activity via estrogen-receptor or non-receptor mediated pathways. Sulfoconjugated estrogen fails to bind estrogen-receptor (ER). High estrogen is a known carcinogen in postmenopausal women. Reports reveal a potential redox-regulation of hSULT1E1/E2-signalling. Further, oxidatively-regulated nuclear-receptor-factor 2 (Nrf2) and NFκβ in relation to hSULT1E1/E2 could be therapeutic-target via cellular redox-modification.

**Methods:**

Here, oxidative stress-regulated SULT1E1-expression was analyzed in human breast carcinoma-tissues and in rat xenografted with human breast-tumor. Tumor and its surrounding tissues were obtained from the district-hospital. Intracellular redox-environment of tumors was screened with some in vitro studies. RT-PCR and western blotting was done for SULT1E1 expression. Immunohistochemistry was performed to analyze SULT1E1/Nrf2/NFκβ localization. Tissue-histoarchitecture/DNA-stability (comet assay) studies were done.

**Results:**

Oxidative-stress induces SULT1E1 via Nrf2/NFκβ cooperatively in tumor-pathogenesis to maintain the required proliferative-state under enriched E2-environment. Higher malondialdehyde/non-protein-soluble-thiol with increased superoxide-dismutase/glutathione-peroxidase/catalase activities was noticed. SULT1E1 expression and E2-level were increased in tumor-tissue compared to their corresponding surrounding-tissues.

**Conclusions:**

It may be concluded that tumors maintain a sustainable oxidative-stress through impaired antioxidants as compared to the surrounding. Liver-tissues from xenografted rat manifested similar E2/antioxidant dysregulations favoring pre-tumorogenic environment.

## Background

Breast cancer is one of the most devastating and common cancers in women worldwide [1]. The global occurrence rate of breast cancer depends on the locations and geographical variations [2]. The rate varies as follows; 27 per 100,000 in Middle Africa and East Asia to 92 per 100,000 in Northern America [3]. In the year 2008, 8 million deaths were recorded as a result of malignant breast cancers, and that figure is assumed to be 11 million by 2030 [4]. Report suggests that, with the increasing of average life-span in developed countries, the incidence of this disease among older people is increasing [5].

Resources and health beneficiaries are one of the major factors of the disease incidence and mortality. Data suggest that high-resource countries have been declining the mortality rate whereas incidence and mortality is in increasing order in low-resource countries [6]. Regarding the causative factors of the disease pathogenesis it may be explained that genetic risk and gene-environment interaction with nutritional status and body compositions have strong role. Genome-wide association studies have identified a number of genetic susceptibility loci in breast cancer risk [7]. Reports reveal that mutations in the BRCA 1 and BRCA 2 tumor suppressor genes are significantly associated with the development of breast and ovarian cancer by the age of 70 [6]. There are different causes that include BRCA1, BRCA2, HER2 neu mutation [8] and causes like elevated ER expression [9]. Estrogen has been regarded as one of the most effective cause in breast cancer [10]. Both endogenous and exogenous estrogen has been already declared as human carcinogen [11] in pre and post-menopausal women [12]. This is also supported by studies in animal models [13]. Estrogen mediates its function through nuclear estrogen receptors such as ERα and ERβ. E2-ER complexes bind directly on DNA and influence the rate of several gene expressions [14]. E2-ER complex also activates protein kinases via non-genomic pathway [15]. Both genomic and non-genomic estrogen activity leads to cellular proliferation and growth during estrous cycle, mammary gland development, and ovarian development. This suggests that E2 may also function at cellular and molecular level via estrogen receptor (ER) independent pathway. So, beside ER involvement, E2 level alone can be a potent determinant of cellular transformation. Post-menopausal women are at higher risk of mammary and other gynaecological cancers. Estrogen in postmenopausal women performs few functions like maintenance of skin and bone density, improvement of collagen content and its quality [14]. Studies report that one-fourth of breast cancer patients carrying BRCA1 mutation were ERα+ by nature [16]. So, rest of the patients carry ERα unrelated disease mechanism. But both groups of patients manifest higher levels of estrogens. These data suggest that estrogen is the most important factor in breast cancer initiation and progression. Studies also report that malignant transformation of certain type of ovarian carcinoma is induced by estradiol signals [17].

Monitoring and targeting estrogen synthesizing and regulatory protein may control estrogen malfunction. Studies report that sulfonated estrogens have no ER binding affinity whereas desulfation of estrogen sulfates may contribute to high levels of active estrogen in target tissues [18]. In this regard, estrogen sulfotransferase (SULT1E1), an important member of the of steroid-sulfotransferases super family, sulfonate estrogen into biologically inactive estrogen sulfates [19]. The binding affinity of estradiol towards the estrogen receptor (ER) is about twice as high as that of estrone, while sulfoconjugates of E2 and E1 show no binding activity towards ER [20] and eventually no estrogenic activity. SULT1E1 activity is significantly declined during the onset of breast carcinogenesis [21]. A correlation between SULT1E1 and the carcinogenesis of estrogen-dependent cancers has been noticed. This is important that the level of SULT1E1 expression is inversely correlated with malignancy in breast cancers [22]. Induction of intra-tumoral SULT1E1 and reduction of estrogen concentration by TM208 contributes to the anti-breast cancer action [23].

Earlier studies suggested a potential oxidative inhibition mechanism for human SULT1E1 through Cys83 redox modification. Cys83 is located in the active site of SULT1E1 and its thiol (–SH) remains in direct contact with the substrate E2. To the best of our knowledge, oxidative regulation of human SULT1E1 in human breast cancer tissue has not been reported yet. Therefore, the aim of this work is to study the role of SULT1E1 in breast cancer tissue, its regulation and its expression. This study also aimed to correlate oxidative stress status and SULT1E1 expression in breast cancer in order to find whether oxidative stress influences SULT1E1 in breast carcinoma. Not only the redox regulation of SULT1E1 but a member of important antioxidant enzyme like SOD, catalase, and GPx has been reported to be modulated in different disease condition. Role of Catalase, SOD, and GPx are directly related to cancer. The comparison of redox regulation of these enzymes in live human breast cancer and its comparison to the corresponding surrounding tissue have not been demonstrated earlier.

The redox environment is associated with breast cancer pathogenesis and metastasis. And in this process roles of oxidatively-regulated nuclear-receptor-factor 2 (Nrf2) which is also a regulator of SULT1E1 and nuclear factor kappa-light-chain-enhancer of activated β cells (NFκβ) have been investigated. Here we have extensively studied the redox regulation of E2 metabolising enzyme SULT1E1, in vivo condition and also in several in vitro models. Moreover, human breast cancer tissue xenografted mouse model has been utilized to characterize the pre-tumorigenic conditions.

## Methods

### Ethical clearance and fulfilments of other regulatory affairs

This is to state that the present study was carried out in accordance with the National Institutes of Health, USA guidelines and the institutional ethical concerns, relevant guidelines and regulations were maintained throughout the investigation. This is to confirm that all experimental protocols were approved by the institutional (Oriental Institute of Science and Technology) Ethics Committee (oist/EC/hu/bt/16/). This is also to state that informed consent was obtained from all participant individuals who were at their post-menopausal age.

Female Wistar rats were purchased from a small-animal firm house (govt. registered) that follows all ethical norms and maintain requisite regulatory affairs. The firm house is a Government accredited [CPCSEA-Committee for the Purpose of Control and Supervision of Experiments on Animals: Reg. no. 1A2A/PO/BT/S/15/CPCSEA  (http://cpcsea.nic.in/Auth/index.aspx)] organization under the Dept of Animal Husbandry and Dairy, Ministry of Agriculture and Farmer’s Welfare, Govt. of India. For all animal experiments, proper permissions were obtained the Institutional (Oriental Institute of Science and Technology) Review Board.

### Inclusion and exclusion criteria

#### Inclusion criteria

Patients only with breast carcinoma were included. Tumors were collected only from those patients who were undergoing mastectomy. And only those patients were included where there was a large gap of time between chemotherapy and mastectomy.

#### Exclusion criteria

Women suffering from endometriosis, pelvic inflammatory disease, tuberculosis, or any kind of liver and kidney disease, ovarian cancers, Poly cystic ovarian syndrome, colon carcinoma, lung carcinoma, pregnancy, menstruation or any other infective disease like HIV, HPV, HCV and Hepatitis B were all excluded.

### Details of the participants (Table 1)

Detailed information about the patients who donated their tumor samples has been provided in Table 1. Table 1 informs about patients livelihood status, nutritional status, grade of the disease, tumor size and the details regarding the inclusion of the lymph nodes and metastasis.

**Table 1:** Details of the breast cancer patients

SN	Age	Livelihood status	Nutritional Status	Cancer grade	Disease description
1	60	Rural	Ethnic food Conserved food Less fast food Diet with less protein and more carbohydrate Low socio-economic status	IIIB	T4—65 mm; N2—6 ALN; M0—no metastasis
2	45	Rural		IIIB	T4—57 mm; N1—3 ALN; M0—no metastasis
3	50	Rural		IV	T4—74 mm; N3—11 ALN; M0—Brain, liver, lung and bones
4	40	Rural		IIIA	T2—33 mm; N2—6 ALN; M0—no metastasis
5	60	Rural		IIIB	T4—68 mm; N1—3 ALN; M0—no metastasis
6	45	Rural		IIIB	T4—59 mm; N1—2 ALN; M0—no metastasis
7	42	Rural		IIIB	T4—61 mm; N1—2 ALN; M0—no metastasis
8	45	Rural		IV	T4—71 mm; N3—10 ALN; M0—no metastasis
9	40	Rural		IIIA	T2—41MM; N2—5 ALN; M0—no metastasis
10	40	Rural		IIIA	T2—44MM; N2—5 ALN; M0—no metastasis
11	40	Rural		IIIB	T4—62; N2—5 ALN; M0—Spread to chest wall
12	38	Rural		IIB	T2—22 mm; N1—2ALN; M0—no metastasis
13	45	Rural		IIIB	T4—70 mm; N2—8 ALN; M0—no metastasis
14	48	Semi-urban		IIIB	T4—66 mm; N2—7 ALN; M0—no metastasis
15	60	Rural		IIIB	T4—78 mm; N2—8 ALN; M0—no metastasis, swelling and ulceration
16	45	Rural		IIIB	T4—73 mm; N2—9 ALN; M0—no metastasis
17	45	Rural		IV	T4—80 mm; N3—12 ALN; M0—Brain, liver, lungs
18	40	Rural		IIIA	T1—18 mm; N0—No ALN; M0—no metastasis
19	40	Semi-urban		IIA	T1—15 mm; N0—No ALN; M0—no metastasis
20	27	Rural		IIIA	T2—39 mm; N1—2 ALN; M0—no metastasis
21	50	Rural		IIIA	T2—32 mm; N1—2 ALN; M0—no metastasis
22	38	Rural		IIB	T1—20 mm; N1—1 ALN; M0—no metastasis
23	45	Rural		IIIB	T4—58 mm; N2—5 ALN; M0—no metastasis

*T* tumor, *N* lymph nodes, *ALN* axillary lymph nodes, *M* metastasis

### Sample collection

The study was conducted in Oriental Institute of Science and technology and a total of 23 breast tumor samples were obtained from local District Medical College and Hospital with proper ethical clearance. Breast tumors are diagnosed clinically; breast cancers were classified on the basis of TNM [The extent of the tumor (T), the extent of spread to the lymph nodes (N), and the presence of metastasis (M)] staging and grades. In some cases down staging of cancer with chemotherapy was done prior to surgery and samples were collected. In this regard this is to mention that tumor samples and corresponding surrounding tissues were collected separately soon after surgery and stored at − 20 °C. A small part of the tissue was also stored in formalin for histology and immunohistochemistry. We sincerely thank Dr. Guangping Chen of Department of Physiological Sciences of Oklahoma State University for providing the primary antibody against rSULT1E1 and hSULT1E1. Nrf-2 (PB9290) and NFkB (PB9149) Antibodies were purchased from BOSTER BIOLOGICAL TECHNOLOGY, CO LTD, 3942B Valley Avenue, Pleasanton, CA 94566.

### Animal selection and treatment

To generate the xenograft model we used Wistar strain. Female rats within the age of 3 to 4 weeks (85–95 g) were acclimatized for 10 days under 12 h light–dark cycle, 25 °C ± 2 °C temperature, 50–70% humidity in the institutional animal house. Animals were fed with a standard pellet diet (Hindustan Lever Ltd, Mumbai, India) and water ad libitum. Studies were carried out, abiding all the guidelines National Institutes of Health, USA and the institutional ethical concerns were also strictly maintained throughout the investigation. Animals were randomly distributed in 2 groups with 5–9 animals in each group. The Group-I animals were kept as control, Group-II animals were implanted with single cell preparation of breast tumor tissue (stage IIB) in the inguinal mammary fat pad area and were fed with 2.5 mg/500 μl of 17β-estradiol (E8875 SIGMA) once in a week for 4 months [24, 25]. This dose and schedule served the establishment of pre-tumorigenic condition. On the day of sacrifice, animals were experienced cervical dislocation (7.30 a.m.) and initially their blood was collected using a disposable syringe (21-gauge needle), serum was separated from the collected blood samples. The liver tissue was carefully collected and stored at − 20 °C for experimental purposes.

### Cytosol preparation

Breast tumor and the corresponding surrounding tissues were homogenized (30% w/v) in the ice-cold phosphate buffer (0.1 mol/l, pH 7.4) and the homogenate was centrifuged at 10,000 rpm at 4 °C for 30 min. The supernatant (cytosol) was collected and stored at − 20 °C in different aliquots for further assays.

### Estimation of malondialdehyde (MDA) levels

The cytosol was used for the estimation of MDA. The MDA assay was conducted following the protocol as in Buege and Aust [26] with a slight modification. To chelate iron and reduce its interference in peroxidation reaction of unsaturated fatty acid, 1 mM EDTA was used in the reaction mixture. To reduce the interference caused by a yellow-orange colour produced by some carbohydrates, the reaction mixture was heated at 80 °C instead of 100 °C. Finally, the MDA was measured and calculated utilizing the molar extinction coefficient of MDA (1.56 × 10^−5^ cm^2^/mmol).

### Estradiol estimation by ELISA

The estradiol in tumor, its corresponding surrounding tissue and plasma of the same patient was determined by combined ELISA and spectrophotometry. Estradiol ELISA kit (Lilac, India) used was based on the principle of a solid phase enzyme-linked immunosorbent assay. Absorbance was measured spectrophotometrically at 450 nm.

### Estimation of non-protein soluble thiol (NPSH)

The NPSH in tumor and corresponding surrounding tissue homogenates (prepared in 0.1 M phosphate buffer, pH 7.4) were determined by the standard DTNB (5,5′-dithiobis-2-nitrobenzoic acid) method with a slight modification. In brief, the protein was precipitated by trichloroacetic acid and clear cytosol was added to 0.1 M sodium phosphate buffer containing 5 µM DTNB. The level of NPSH was determined against a GSH standard curve [27].

### Assay of super oxide dismutase (Cu–Zn, SOD1), catalase and GPx activities

#### SOD activity by zymogram

A tablet of nitro blue tetrazolium (NBT) was dissolved in 30 ml of water and the non-denaturing (10%) acrylamide gel was soaked with it for 30 min with shaking. The gel was then shaken in 40 ml SOD solution 0.028 M tetramethylethylenediamine (TEMED), 2.8 × 10–5 M riboflavin, and 0.036 M potassium phosphate at pH 7.8) for 15 min. The soaked gel was placed on a clean acetate sheet and illuminated for 5 to 15 min. The gel became purple except at the position containing SOD1. The gel was scanned when the maximum contrast between the band and background has been achieved [28].

#### Catalase activity was measured by an in gel zymogram assay

A non-denaturing gel (8%) loaded with 50 μg of protein was incubated in 0.003% H_2_O_2_ for 10 min followed by staining with 2% ferric chloride and 2% potassium ferricyanide. The gel became greenish blue except at the position having the catalase. The gel was scanned when maximum contrast between the band and the background was obtained [28].

#### GPx activity assay by in gel zymogram assay

A non-denaturing gel (8%) loaded with 100 μg of protein was incubated in GSH followed by cumene hydroperoxide for 10 min and then stained with 2% ferric chloride and 2% potassium ferricyanide. The gel became greenish blue except at the position containing the GPx. The gel was scanned when maximum contrast between the band and the background was obtained [28].

#### Enzyme activity modification by redox change

RBC extracts were prepared by a standardised method. Equal amounts of protein were incubated with different concentration of H_2_O_2_, H_2_O_2_ + β-ME and only β-ME for 2 h at RT. After the incubation period of 2 h activity assay for SOD, catalase and GPx was performed by zymogram activity assay method.

### Western blot analysis of estrogen sulfotransferase (SULT1E1)

Western blot was conducted following an earlier standardized protocol as in Maiti et al. [29] with a slight modification. A 12% denaturing gel was loaded with 25 μg of protein and electrophoresis was done at 100 v for 3 h, transfer was done at 100 v for 2 h. The membrane was washed and incubated in primary and secondary antibodies as mentioned in the protocol. Brown coloured bands were developed by using diaminobenzidine (DAB).

### RT-PCR

Whole RNA was isolated from surrounding and tumor tissue obtained from breast cancer patients and from E2 treated and untreated female rat livers. A reverse transcription PCR was performed using 1 μg of whole RNA and Qiagen one step RT-PCR kit following the protocol provided in the kit.

### Comet assay

The alkaline Comet assay was performed according to the standard method with minor modifications [30]. A total of 75 µl of low melting point agarose (0.6%) in PBS at 37 °C was added to a 25 µl of cell suspension (105 cells). The mixture was then dropped onto a microscope slide pre-coated with 1% agarose. After solidification the slides were immersed in ice-cold lyses buffer for 1 h at 4 °C. Slides were then incubated at 37 °C for 45 min. Slides were incubated in alkaline electrophoresis buffer (0.3 M NaOH and 1 mM EDTA) for 25 min. After incubation, electrophoresis done for 30 min at 25 V and the current was adjusted to 300 mA. Slides were neutralized with PBS and stained with a 10 mg/ml ethidium bromide. Slides were read using a fluorescence microscope (Nikon, Eclipse LV100 POL), with the VisComet (ImpulsBildanalyse) software.

### Histoarchitecture studies of cancerous tissues

Cancerous growth in breast tissue and its surrounding tissues were embedded in paraffin, serially sectioned at 5 μM by an automated cryostat slicing machine (Leica Biosystems), stained with eosin and haematoxylin (Harris), and observed under a microscope (Nikon, Eclipse LV100, magnification 10× and 20×) to study the tissue histoarchitecture.

### Immunohistochemistry analysis for NFκβ and Nrf2

Tumor and its corresponding surrounding tissues were embedded in paraffin, serially sectioned at 5 μM by an automated cryostat slicing machine (Leica Bio systems). Sections were deparaffinised by baking at 60 °C followed by xylene treatment, downgraded alcohol and water. Slides were washed with PBST containing 1% casein for 10 min, tissue sections were incubated with 5% casein for 30 min for preventing non-specific binding followed by overnight incubation in primary antibody NFκβ and Nrf2 in 1% casein PBST, washed with 1% PBST and incubated in 1% casein PBST containing secondary antibody for 1 h, washed with 1% PBST followed by water and stained with chromogenic substrate DAB for 3 min and then washed with water. Slides were fixed with mounting medium and observed under a microscope (Nikon, Eclipse LV100, magnification 20×) to study the SULT1E1 expression and localization.

### Density analysis of Western blot band

ImageJ software was used for this analysis. SULT1E1, Nrf2 and NFκβ expression in tumor and its corresponding surrounding tissues were studied by immunohistochemistry (IHC). The percentage of binding of the specific antibody was analysed by IHC scoring in three slides from independent study result in tissues from different individuals. The scoring was made in terms of percentage of the area being covered by the DAB colour (brown) and its intensity. To calculate the intensity of the % of area we obtained the median value of the signal in the IHC picture by using the measurement command and the analytical protocol of the program.

### In-vitro induction of SULT1E1 and Nrf-2 in rat hepatocytes

#### Preparation of single cell suspension of rat hepatocyte

1 g of rat liver was cut into tiny pieces and scrapped through nylon mesh in DBSS buffer. The homogenate was centrifuged at 120×*g*. The pellet was dissolved and washed with L-15 media (1500 mg/l d-glucose, 20% FBS, 1% penicillin and streptomycin, 1% Glutamine). Cells were finally kept in 2 ml of L-15 media. 650 µl of stock cell media was added to petri plates containing 9350 µl of L-15 media.

#### Drug treatment

Dexamethasone was added at a concentration of 100 µM. Lansoprazole was added at a concentration of 25 mg per plate. The culture was incubated for 72 h under conventional condition in an incubator maintained at 37 °C.

### Statistical analysis

The statistical analyses were done by using the SPSS for Windows statistical software package (SPSS Inc., Chicago, IL, USA, 2010). Normally distributed data were tested by Kolmogorov–Smirnov test. Baseline continuous-variables and outcome-measures were compared by Students t’ test analysis. Pearson’s correlation study was done to verify the association between different disease causing factors and disease grades.

## Results

### Oxidative stress markers suggests higher free radical burden in the tumor tissue

**Fig. 1:**
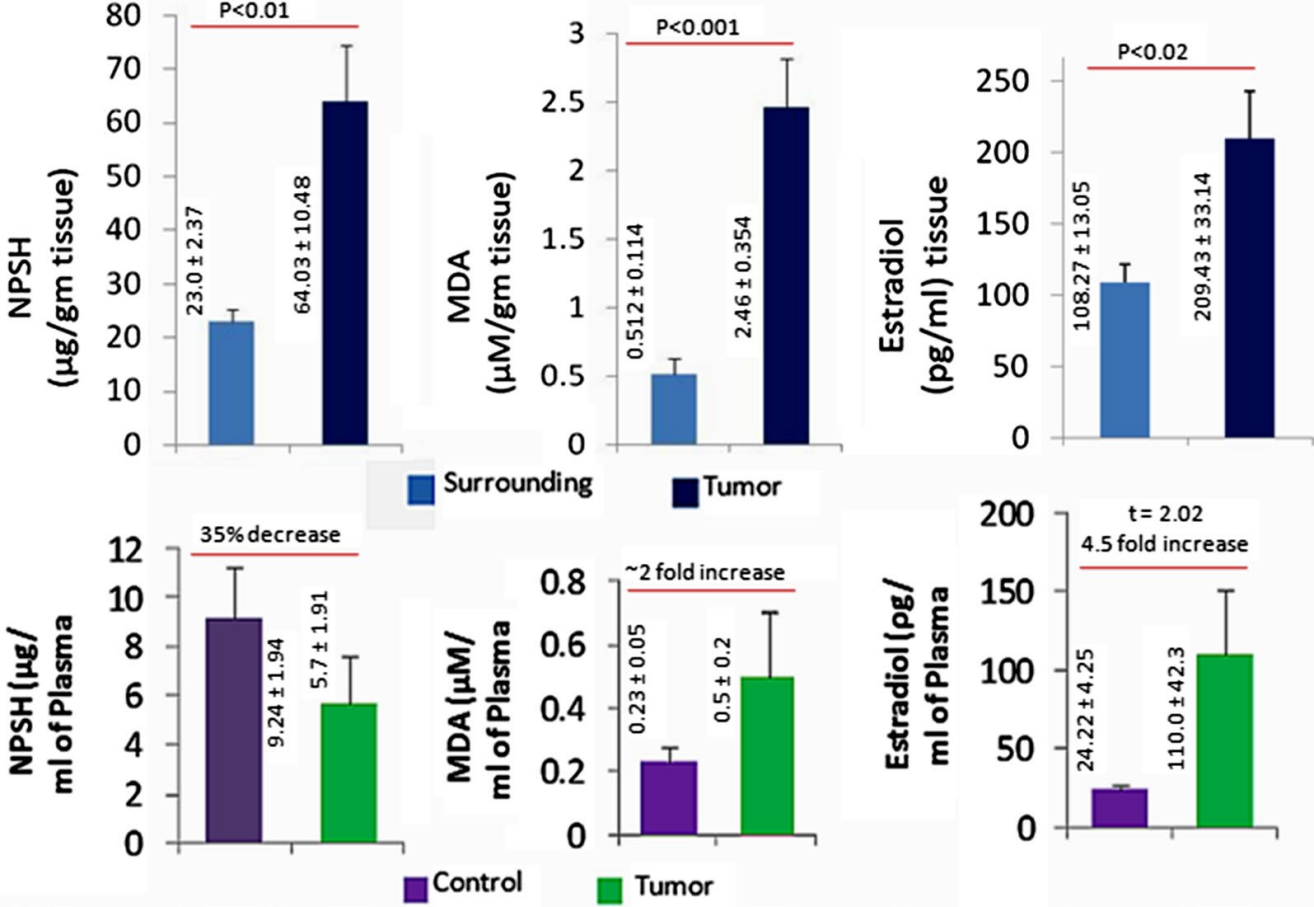
NPSH, MDA and Estradiol level in tumor tissue, its corresponding surrounding region and plasma. Results are mean ± SE (n = 17–23 in different groups). Data of tumor group is compared to the surrounding group (Student ‘t’ test). Levels of significances (p values) are mentioned in the figure

The mean NPSH level in the surrounding and the tumor tissue was not significantly increased in terms of protein, and was found to be 2.99 (± 0.818) in the surrounding and 3.17 (± 0.516) in the tumor tissue (not presented in figure). Whereas, the mean NPSH level in terms of wet weight of tissue was found to be higher in the tumor that was 64.03 μg/g compared to the surrounding μg/g (p < 0.01). The end product of lipid peroxidation, MDA was found to be higher in the tumor 2.46 μM/g as compared to the corresponding surrounding 0.512 μM/g (p < 0.001) (Fig. 1).

### Estradiol level in tumor tissue compared to its surrounding tissue

The average estradiol was found to be higher in the tumor as compared to its corresponding surrounding with mean values 108.27 ρg (± 13.05) in the surrounding it was 209 pg (± 33.14) in the tumor tissues (Fig. 1).

### Alterations in the activities of antioxidant enzymes in the tumor tissue

**Fig. 2:**
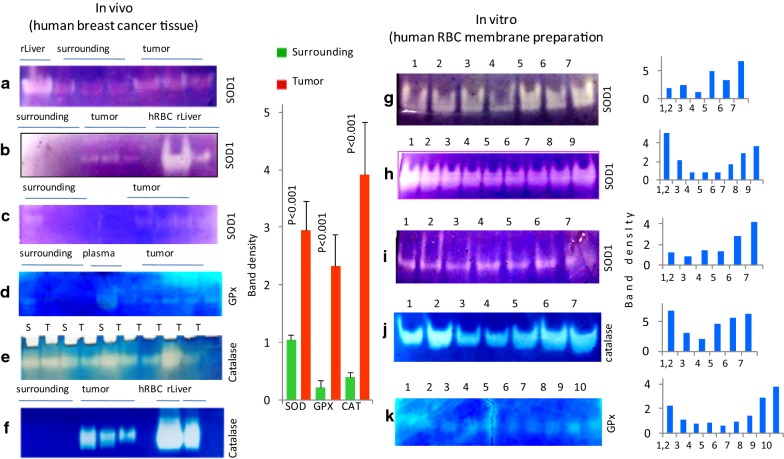
In vivo (left panel) representative zymographic activities of SOD, catalase and GPx activity in tumor and corresponding surrounding tissue (in-vivo). SOD1: **a** 100 µg protein loaded, lane distribution: 1—rat liver; 2, 3, 4—surrounding; 5, 6, 7—tumor; **b** 100 µg protein loaded, lane distribution: 1, 2, 3—surrounding; 4, 5, 6—tumor; 8—human RBC; 9—rat liver. **c** Lane 1, 2, 3, 4—surrounding tissue; 6, 7, 8, 9—tumor tissue. GPx-d: 100 µg protein loaded—lane distribution: 1, 2, 3, 4—surrounding; 5—plasma; 6, 7, 8, 9, 10—tumor. Catalase: **e** 100 µg protein loaded—lane distribution: 1, 3, 5—surrounding; 2, 4, 6, 7, 8, 9, 10—tumor. **f** 100 µg protein loaded—lane distribution: 1, 2, 3—surrounding; 4 to 6—tumor; 8—RBC; 9—rat liver. Densitometry results are mean ± SE (n = 17–23 in different groups). Data of tumor group is compared to the surrounding group (Student ‘t’ test). Levels of significances (p values) are mentioned in the figure. In vitro SOD, catalase and GPx activity modification by redox change with H_2_O_2_ and β-ME (in-vitro, right panel). **g** SOD1: 1, 2—control; 3—0.5 M H_2_O_2_; 4—0.1 M H_2_O_2_; 5—100 µM BM; 6—10 µM BM; 7—1 µM BM. **h** SOD1: 125 μg of human RBC protein loaded, Lane: 1, 2—control; 3—100 mM H_2_O_2_; 4—1 M H_2_O_2_; 5—1 M H_2_O_2_ + 1 nM BM; 6—1 M H_2_O_2_ + 0.1 μM BM; 7—1 M H_2_O_2_ + 1 μM BM; 8—1 M H_2_O_2_ + 10 μM BM. **i** SOD1: 1, 2—control; 3—0.1 M H_2_O_2_; 4—0.01 M H_2_O_2_; 5—10 μM BM; 6—1 μM BM; 7—0.1 M H_2_O_2_ + 100 μM BM. **j** Catalase: 1, 2—control; 3—500 mM H_2_O_2_; 4—1 M H_2_O–; 5—0.1 μM BME + 1 M H_2_O_2_; 6—1 μM BME + 1 M H_2_O_2_; 7—10 μM BME + 1 M H_2_O_2_. **k** GPx: 1, 2, 3—control; 4—100 μM H_2_O_2_; 5—500 mM H_2_O_2_; 6—1 M H_2_O_2_; 7—1 M H_2_O_2_ + 1 μM β-ME; 8—1 M H_2_O_2_ + 10 μM β-ME; 9—1 M H_2_O_2_ + 100 μM β-ME; 10—1 M H_2_O_2_ + 1 mM β-ME

The antioxidant enzyme superoxide dismutase (SOD) activity was found to be higher in the tumor as compared to its surrounding (Fig. 2, SOD1). The catalase activity was found to be stronger in the surrounding as compared to the tumor with exceptional, where tumors had strong catalase activity (Fig. 2, Catalase). GPx was elevated in the tumor as compared to the surrounding (Fig. 2, GPx).

### Alterations in antioxidant enzymatic activities by redox modification

Equal amounts of RBC protein were incubated with different concentration of H_2_O_2_, H_2_O_2_ + β-ME and only β-ME for 2 h at RT. The activity of SOD, catalase and GPx were impaired in presence of H_2_O_2_ while the activity was improved in presence of H_2_O_2_ + β-ME and only β-ME (Fig. 2).

### hSULT1E1 protein expressions in breast tumor tissues

**Fig. 3:**
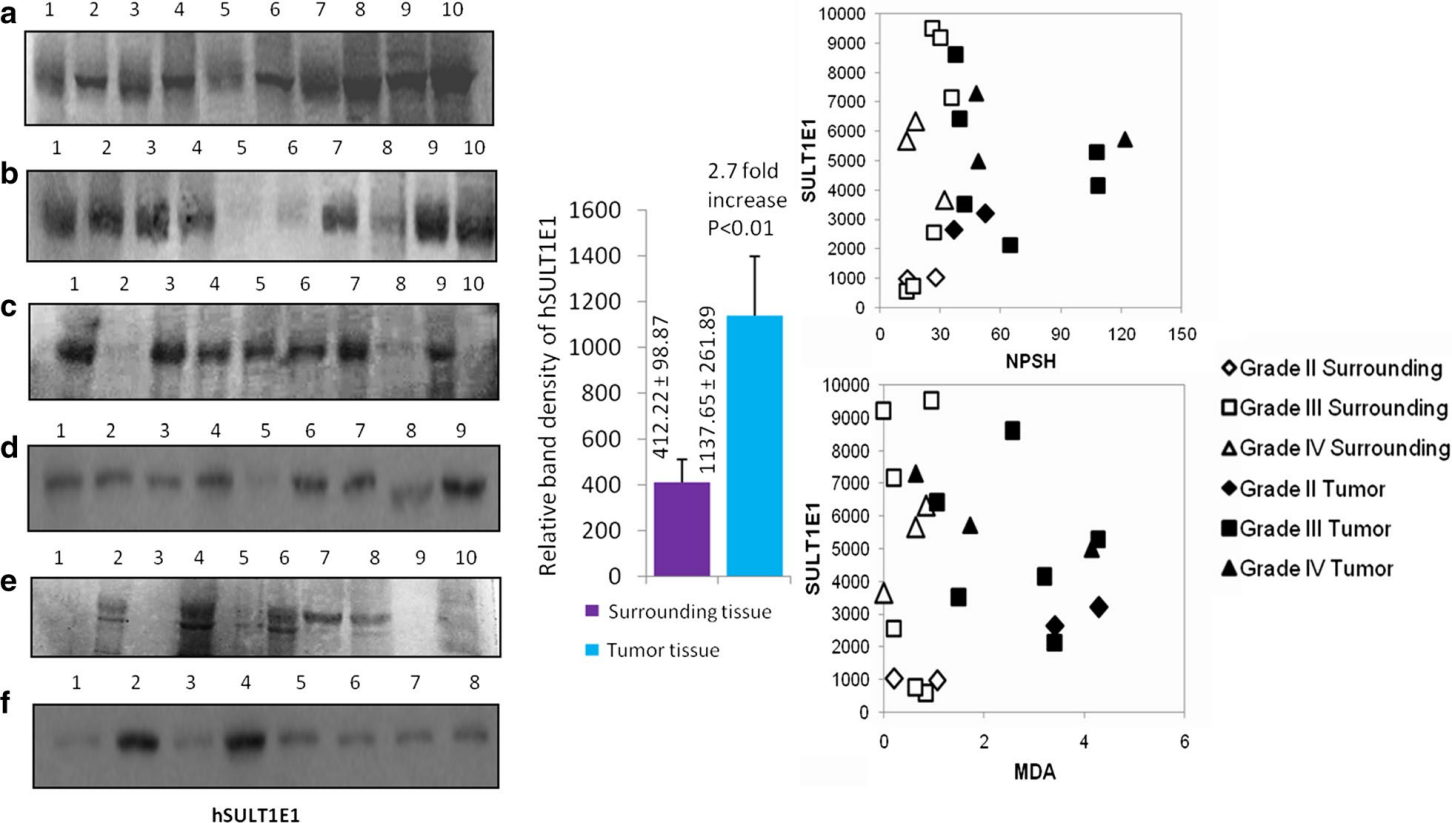
SULT1E1 expression in tumor and its corresponding surrounding tissue. Left panel: **a** 1S,2T-IIIB(60); 3S,4T-IIIB(45); 5S,6T-IV(45); 7S,8T-IIIA(40); 9,10-R.L. **b** 1S,2T-IIIB(60); 3S,4T-IIIB(45); 5S,6T-IIIB(42); 7S,8T-IV(45); 9S,10T-IIIA(40). **c** 1S,2T-IIIA(40); 3S-IIIA(40), 4T-IIIB(27); 5-IIB(38); 6-IIIA(40); 7-IIIB(40); 7S-IIIB(45); 8T-IIIB(42); 9S-IIIB(45); 10-II(40). **d** 1S,2T-IIIB(60); 3S,4T-IIIB(45); 5S,6T, 7T-IV(45); 8S,9T-IIIA(40); 9S,10T-II(40). **e** 1S,2T-IIIB(27); 3S,4T-IIIA(50); 5S,6T-IIB(38); 7S,8T-IIIB(45); 9-empty; 10-Marker: 1S,2T-III(49), 3S,4T-IV(52), 5S,6T-(II), 7S,8T-II(47). (Some of the sample has been run in duplicate or triplicate). Middle panel: densitometry data (mean ± SE) and their statistical values (Student‘t’ test) are presented as the bar diagram. Levels of significances (p values) are mentioned in the figure

An irregular pattern of SULT1E1 expression was noticed, in some of the cases including IIIB, IIIA, SULT1E1 was found to be highly expressed in the tumor tissue compared to their corresponding surrounding tissues (Fig. 3). And in some cases including stages IIIA, IIIB, IIIC and IV, SULT1E1 expression was higher in the surrounding compared to its corresponding tumors (Fig. 3).

### rSULT1E1 protein expressions in rat liver

**Fig. 4:**
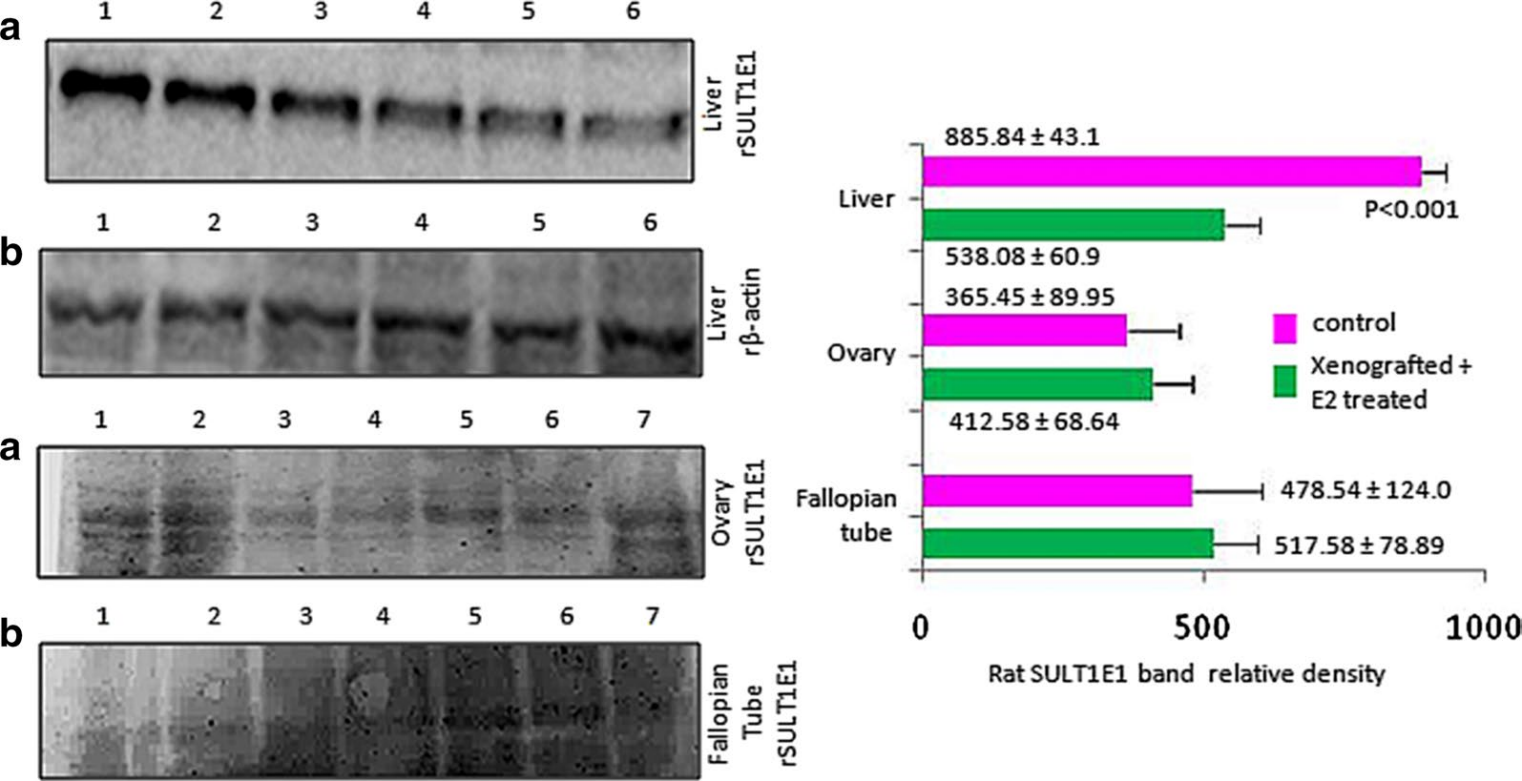
Representative Western blot picture of SULT1E1 expression in liver, ovary and fallopian tube tissues of xenografted-E2 rat and corresponding control. **a** 1, 2, 3—control; 4, 5, 6—Xeno-E2 (liver); **b** liver Β-actin; **c** 1, 2, 3—control; 4, 5, 6—Xeno-E2 (ovary); **c** and **d** ovary and fallopian tube respectively, 1, 2, 3—control; 4, 5, 6, 7—Xeno-E2. Densitometry data are the mean ± SE (n = 5–9 in each group). Data of treated group is compared to the corresponding vehicle treated group (Student ‘t’ test). Levels of significances (p values) are mentioned in the figure

The protein level of SULT1E1 expression was found to be higher in the untreated rat liver compared to those treated with estradiol. The SULT1E1 expression in the ovary and fallopian tubes of the E2 treated animals was found to be higher as compared to the untreated animals (Fig. 4).

### SULT1E1 mRNA expression in breast cancer patients and E2-xenograft rat model

**Fig. 5:**
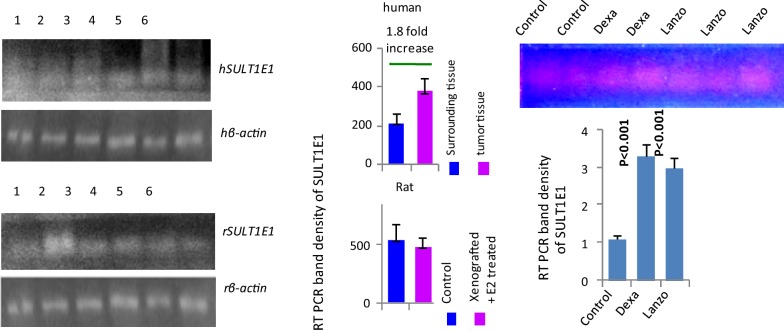
SULT1E1 mRNA expression in breast tumor, its corresponding surrounding and in liver of xenografted-E2 rat. Human: **a** 1, 2, 3—surrounding; 4, 5, 6—tumor. Rat: **b** 1, 2, 3—control; 4, 5, 6—Xeno-e2. Rat: **c** 1, 2—control; 3, 4—dexamethasone; 5, 6, 7—lansoprazole. Densitometry data are the mean ± SE. Levels of significances (p values) are mentioned in the figure

SULT1E1 mRNA was high in tumors and less in surrounding tissue (Fig. 5). SULT1E1 mRNA expression in liver was inhibited in the E2 treated female rats compared to the untreated animal livers (Fig. 5).

### Comet assay with tumor tissues

**Fig. 6:**
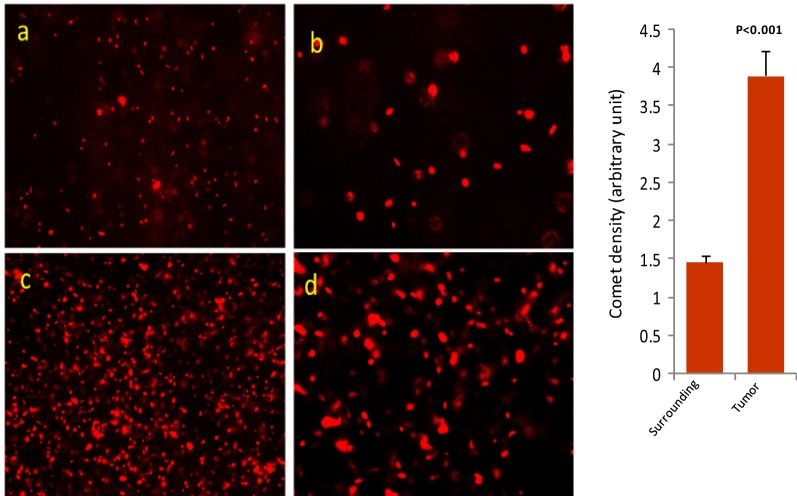
Comet assay to analyse DNA of breast tumor and its corresponding surrounding tissue. **a**, **b** Surrounding; **c**, **d** tumor. Densitometry data are the mean ± SE of 50 analysis of each surrounding and tumor. Levels of significances (p values) are mentioned in the figure

The comet assay reveals high cellularity and unorganized cells in the breast tumor tissues (Fig. 6c, d) as compared to the corresponding surrounding tissue (Fig. 6a, b). Both the tumor and the surrounding tissue shows integrated DNA but the tumor tissue shows some larger/expanded DNA materials. A densitometry analysis is done to compare the surrounding with the tumor.

### Histoarchitecture results of tumor tissues

**Fig. 7:**
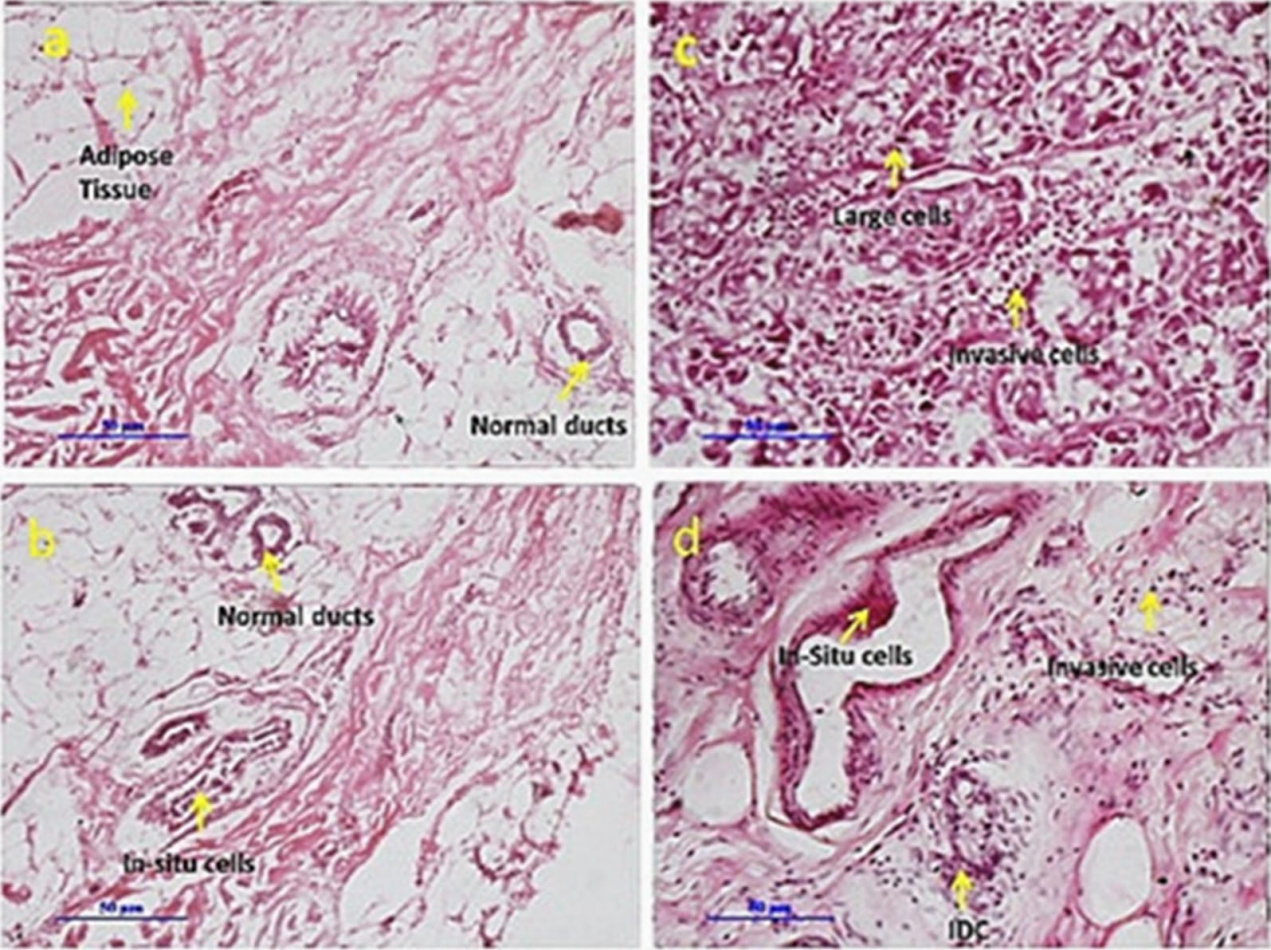
HE-staining of breast tumor and its corresponding surrounding. **a**, **b** Surrounding; **c**, **d** tumor

Histological studies of ductal breast carcinoma of different stages including grade III and grade IV are presented in Fig. 7. Slide (a) represents the surrounding tissue of a pleomorphic carcinoma. Slide (c) represents its corresponding tumor which is high-grade variant of invasive ductal carcinoma (IDC). This high-grade carcinoma is associated with areas of necrosis, inflammation and necrosis. In this field there are spindle cells, small round cells, large cells with nucleoli and giant cells with no differentiated ducts present. Highly pleomorphic nuclei and many mitoses were also noticed.

Slide (b) of Fig. 7 represents the surrounding tissue of a micro invasive tumor. Slide (b) shows normal ducts with in situ cells, adipose tissue, and a histoarchitecture which is close to normal architecture. Slide (d) represents stromal micro invasive ductal cancer and shows atypical ductal hyperplasia, ductal carcinoma in situ with micro invasion of cancer cells in the stromal region. Micro invasive ductal carcinoma is typically found to be associated with fibroblast proliferation, collagenisation and focal inflammation. This infiltrative cancer tissue features cellular stroma accompanying small epithelial and ductal structures. Normal duct channels, blood vessels and lymphatic channels were distorted in both the invasive carcinomas.

### Immunohistochemistry of NFκβ and Nrf2 tumor tissues

**Fig. 8:**
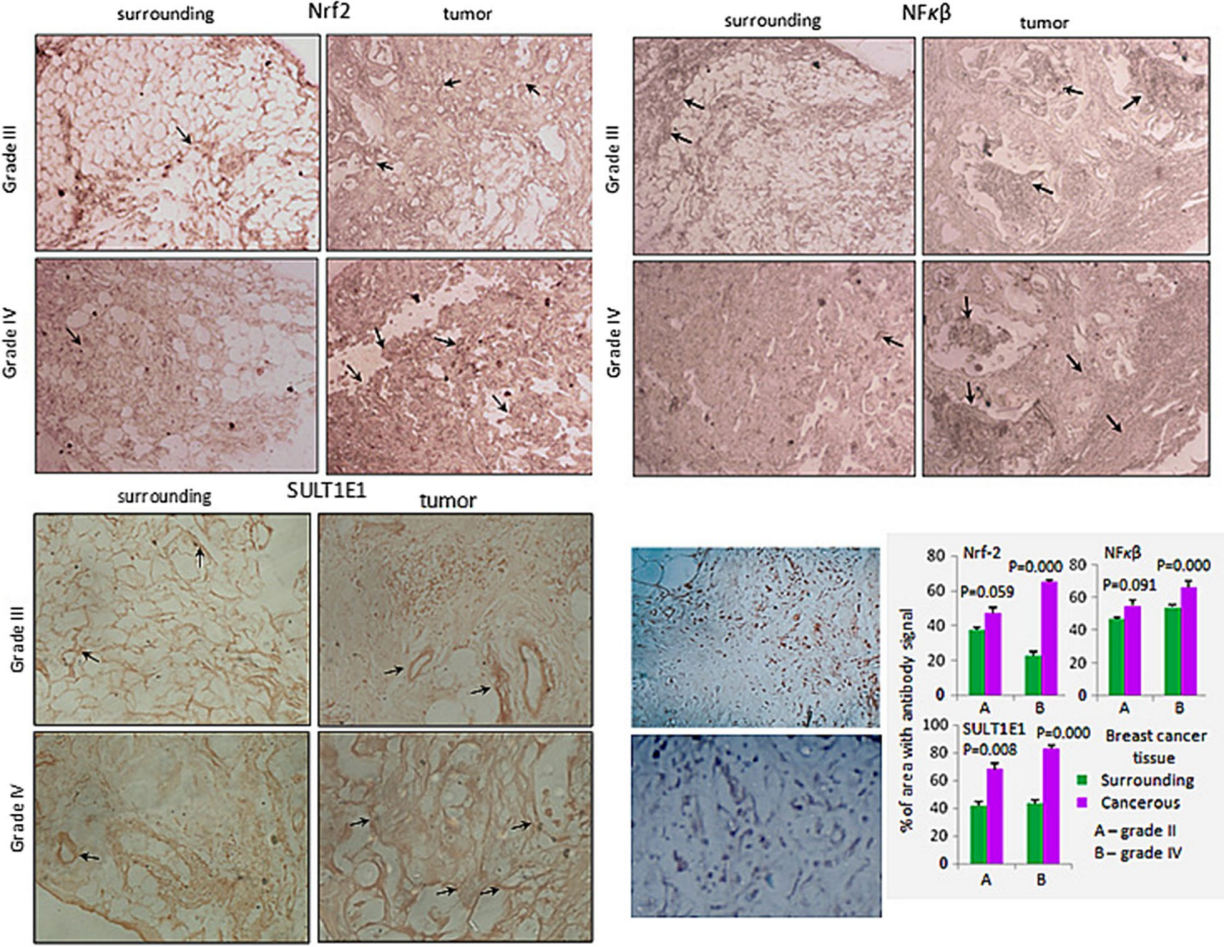
Immuno-histochemistry of Nrf2 and NFκβ in human tumor and corresponding surrounding tissue sample. **a**, **b** Surrounding; **c**, **d** tumor

Immunohistochemistry results show that the tumor and surrounding are positive for NFκβ and Nrf2. The strength of NFκβ and Nrf2 staining in tumor is comparatively much stronger than surrounding. Please refer to the lower right panel for the densitometric analysis data (p < 0.05 and p < 0.001 respectively). The distribution and localization of NFκβ and Nrf2 was noticed in the tumor tissue was much more in stroma and the ductal lining of the tumor with comparison to their corresponding surrounding tissues. Tumor tissue was darkly stained for both NFκβ and Nrf2 compared to the surrounding suggests increased expression and non-uniform distribution of NFκβ and Nrf2 in tumor (Fig. 8).

### In-vitro induction of SULT1E1 and Nrf2 in rat hepatocytes by dexamethasone and lansoprazole

With an aim to verify whether Nrf2 induction will upregulate SULT1E1 expression we incubated rat liver cells in lansoprazole which is a known Nrf2 inducer and a direct SULT1E1 inducer known as dexamethasone. Lansoprazole is an established antioxidative stress inducer and eventually Nrf2 induction. These results clearly explain that induction of Nrf2 does induce SULT1E1 as evident in lansoprazole group. The SULT1E1 induction was comparatively low in lansoprazole group as compared to dexamethasone. The results suggest that Nrf2 induction does induce SULT1E1 expression (Fig. 5c).

### Statistical analysis

Breast tissue SULT1E1 expressions in both grades-II and grade-IV disease conditions were noticed to be significantly correlated with tumor expressions of Nrf-2 (Table 2). NFκβ expressions were found to be significantly correlated with Nrf-2 and SULT1E1 expressions in the tumor tissues (Table 2).

**Table 2:** Statistical analysis of Pearson’s correlations amongst Nrf-2, NFκβ and SULT1E1 in two different diseases conditions in the tumor tissues

	NFκβ/Gr IV	SULT1E1/Gr II	SULT1E1/Gr IV
Nrf-2/Gr II		r = 0.998/p = 0.038	
Nrf-2/Gr IV	r = 0.993/p = 0.075		r = 0.997/p = 0.049
NFκβ/Gr IV			r = 0.999/p = 0.025

No correlations were noticed in the surrounding tissues. Protein expression was studied by immunohistochemistry and ImageJ software was utilized for their density analysis. Results were analyzed from three independent investigations

In case of Disease Grade II, surrounding NPSH, MDA and SULT1E1, Tumor NPSH, MDA and SULT1E1 and the percentage of differences of these three parameters are found to be significantly correlated with each other (Table 3). In case of tumor tissues SULT1E1 is positively correlated with NPSH (p < 0.023) and MDA (p < 0.042). In case of surrounding tissue SULT1E1 is negatively correlated with MDA (p < 0.025). When surrounding SULT1E1 is compared to that in tumor tissue a highly significant and positive correlation is noticed (p < 0.005) (Table 3).

**Table 3:** Statistical analysis of Pearson’s correlations amongst different oxidative-stress parameters and SULT1E1 expressions in three different diseases conditions in the tumor/surrounding tissues

Grade	S_SULT1E1	T_NPSH	T_MDA	T_SULT1E1	PD_NPSH	PD_MDA
II	All parameters of **S** and **T** correlated at p < 0.05 to p < 0.001 level					
III						
S_NPSH						
r	0.761	− 0.655	− 0.435	0.807	− 0.865*	0.456
P	0.079	0.158	0.389	*0.052*	*0.026*	0.363
S_MDA						
r	− 0.257	0.948**	0.925**	− 0.417	0.816*	− 0.17
P	0.623	*0.004*	*0.008*	0.41	*0.048*	0.747
T_NPSH						
r	− 0.152	1	0.798	− 0.322	0.877*	− 0.362
P	0.774		0.057	0.533	*0.022*	0.481
IV						
S_NPSH						
r	− 0.893	− 0.281	0.997*	− 0.878	− 0.637	− 0.292
P	0.298	0.819	*0.05*	0.317	0.56	0.811
S_MDA						
r	1.000**	0.683	− 0.855	0.569	0.916	0.691
P	*0.001*	0.522	0.347	0.615	0.263	0.514
T_NPSH						
r	0.683	1	− 0.205	− 0.212	0.919	1.000**
P	0.521		0.868	0.864	0.258	*0.007*

A significant correlation between SULT1E1 expression and MDA and NPSH were noticed in all disease grades

Italic values indicate level of significance p < 0.05

*Correlation is significant at the 0.05 level (2-tailed)

In higher disease condition intra-individual variability is highly increased. It is well established that cancer is a multi-factorial disease and the extent of impairment of different factors varies from individual to individual. At disease grade III, the correlation amongst the experimental parameters decrease. Similar to the disease grade II, surrounding NPSH was found to be positively correlated to the tumor SULT1E1 (p < 0.049). Surrounding MDA was found to positively correlated with the MDA (p < 0.008) and NPSH (p < 0.004) in the tumor sample (Fig. 3).

When the parameters analyzed for correlation testing in the grade IV disease group no significant correlation was noticed amongst most of the parameters due to the excessive high level of variability. At this state individual variability become distinct in terms of their disease pathogenesis and cellular adaptive mechanisms. Here surrounding MDA was noticed to be positively correlated with surrounding SULT1E1. For all these data it can be concluded oxidative stress has an influence on SULT1E1 expression and activities. When all the disease groups are compared together it is noticed that SULT1E1 expressions in the tumor tissues are highly correlated to that of the surrounding tissues (Fig. 3).

## Discussion

Oxidative regulation of E2 function via SULT1E1 modification (at Cys83) is reported [23]. One recent article extensively analyze that not only SULT1E1 but also a large number of E2 metabolizing proteins including sulfatases (STs), formyl glycine forming enzymes are redox regulated. Nevertheless, E2 signalling helper the ER may also be redox regulated [25]. Moreover, the reverse nature SULT1E1 and ST function in E2 metabolism is very delicately favoured during redox regulation procedure [31]. The current manuscript clearly shows the redox regulation of SULT1E1 and other radical-metabolizing enzymes in the pathogenesis of human breast cancer of different stages. This redox regulation is demonstrated to be related to intracellular level of oxidant stress and related gene expressions like Nrf2 and NFκβ. Breast cancer may depend on both genetic and hormonal factors. Epidemiologic and experimental data infers that mainly estradiol (E2) plays a pivotal role in the development and progression of breast cancers by promoting cell proliferation via ER signalling. It initiates mutations that occur as a function of errors during DNA replication [32]. E2 harbours significant number of mutations, ultimately resulting in cancers. Receptor independent effects of E2 may also be involved in the breast carcinogenesis [32]. In animal models E2-administration and in human E2-therapy causes breast cancer which may be prevented by the anti-estrogenic drugs tamoxifen or raloxifene [33, 34]. This might be possible due to altered metabolism of E2 and direct effect of E2-induced stress. Our earlier study showed that E2 administration to human tumor xenografted rat impaired SULT1E1 protein/mRNA expression resulting higher plasma E2 level [35]. Anti-estrogens [36] and other cancer therapeutic drugs [37] have been shown to alter SULT1E1 protein/gene expressions and E2 signalling rate. This suggests that regulations of SULTs have great implications in E2 function. In the current study, the presence of higher in situ estradiol infers either its increased rate of synthesis or its high rate of accumulation in the circulation. It is reported that cellular uptake of estrogen metabolites (E1S) takes place by human organic anion transporting polypeptide 1A2 (OATP1A2) which is up-regulated by the elevated expression of pregnane X receptor (PXR) in malignant tissue [38].

So, strategies to minimize estrogen activities are proved effective measure to treat breast cancers. Earlier reports hypothesise that, women with mutations in estrogen metabolizing enzymes are expected to develop breast cancer [39]. This suggests a major role of estrogen metabolizing enzymes like aromatase, sulfatase, 17β-HSD and SULT1E1 in breast cancer. Aromatase converts circulating androgen, androstenedione into estrone [40]. Sulfatase (STS) hydrolyses circulating estrone sulfate to estrone (E1) [41]. E1 subsequently converted to estradiol (E2) by 17β-HSD type 1 [42] affecting breast cancer cells through ERα and ERβ. However, studies on estrogen metabolizing enzymes have been inconsistent in linking breast cancer risk with their altered expression/activities. SULT1E1 acts at nanomolar concentration of estradiol and SULT1A1 acts at micromolar concentrations of (E2). So, SULT1E1 can inactivate most of this hormone present [43, 44]. Expression of SULT1E1 in MCF-7 cells reduced the response to physiologic concentrations of estradiol and inhibited estrogen-stimulated DNA synthesis and cell proliferation. Elevated E2 level in the tumor (Fig. 1) suggest that SULT1E1 expression is not sufficiently enough for E2 inactivation (Fig. 3) [20]. In-situ inactivation of E2 is possible by SULT1E1 induction and it may be a way to decrease breast cancer risk.

In our study, an increased SULT1E1 expression is noticed in breast tumor samples as compared to their corresponding surrounding tissue and comparatively low in some tumors resulting poor prognosis (Fig. 3) [45]. Tumor expression of SULT1E1 was also found to be positively correlated with ER-β and PR-B, which are associated with an improve prognosis for breast cancer [41]. Studies also report that over-expression of SULT1E1 inhibits proliferation, induce cell apoptosis, suppress angiogenesis, arrest cell cycle in vitro and tumorigenesis in vivo [46]. And this protective function of SULT1E1 was associated with increase in expressions of MMP-2 and MMP-9 in MCF-7 cell based xenograft mouse [47]. Earlier reports from our lab suggested a potential redox regulation of hSULT1E1 through Cys83 modification [29]. It is of worth notify that even in higher SULT1E1 condition adverse response from other stress regulated gene i.e., HIF1α, NFκβ may also promote the disease.

Estrogens are converted into catechol estrogens and during this process ROS are produced [48]. Estrogen quinones are conjugated with glutathione (GSH) both in vivo and in vitro by glutathione transferases [49] which results in high level of DNA protection [44]. In our studies elevated NPSH in tumors (Fig. 1) compared to corresponding surrounding suggests a possibility of thiol conjugation of estrogen metabolites and protecting tumor cells from ROS/drug induced DNA damage. The increase of malondialdehyde in the tumor than their corresponding surrounding tissues (Fig. 1) interprets a high rate of lipid peroxidation resulting from free radicals/H_2_O_2_ and oxidative stress. In human breast cancer cells, extracellular signal-regulated kinase (Erk1/2) which was activated by H_2_O_2_ generated as a by-product during estrogen metabolism increases cell proliferation [20]. Recent data suggest that H_2_O_2_ may cross cellular membranes through specific members of the aquaporin family [50].

ROS generation during estrogen metabolism or other potential mammary carcinogenic factors as evident from MDA results (Fig. 1) was shown to activate the PI3K/Akt signalling pathway [51, 52]. Serine/threonine-specific protein kinase (Akt) activation generates the anti-apoptotic and anti-inflammatory responses in tumor cells, thereby protecting it from drug induced apoptosis or inflammatory apoptosis. The elevated NPSH in tumors (Fig. 1) increases intracellular redox potential and an adaptive protective strategy against ROS dependent inhibitory (apoptotic) signalling. Imbalance in glutathione system induces programmed cell death in tumors. Elevated NPSH supported increased SOD activity in the tumors (Fig. 1) as compared to their corresponding surrounding tissue. This causes a quick conversion of superoxide into H_2_O_2_ and facilitating tumors with low superoxide [53, 54]. Activity of catalase was variable in tumors and surrounding (Fig. 2). Catalase over-expression (CAT3 cells) increased the resistance of cancer cells to drugs inducing oxidative stress, likely by increasing the antioxidant status of cancer cells [55]. Hydroxyl radical attack DNA rapidly due to their high infusibility which results in formation of DNA lesions including oxidized DNA bases, single strand and double strand breaks [56]. These can be novel supporting therapeutic strategies. Low doses of hydrogen peroxide and superoxide stimulate cell proliferation in a wide variety of cancer cell types [57] which is evident from our study where SOD and catalase activity are highly favouring to cancer cells with low oxidative stress. Increased generation of hydrogen peroxide drives the proliferating cells to transit into quiescence [57, 58]. Increased expression of a variety of enzymes like SOD, Cat and GPx (Fig. 2) that contribute to oxygen radical scavenging [58, 59] favours disease pathogenesis by inhibiting the pro-oxidant effect of drugs.

To verify whether redox regulation of antioxidant enzyme is possible or not, human RBC membrane enzymes were analysed. Since, RBC contains very high level of antioxidant enzymes against ROS. SOD, CAT and GPx were incubated with different concentration of H_2_O_2_ and β-ME alone or together. It was noticed that both SOD and CAT activity were reduced in the presence of H_2_O_2_ while the activity was increased by the reducing equivalent like β-ME (Fig. 2). Reversible oxidation of phosphatases and cysteine within the catalytic sites of enzymes may hinder their enzymatic activity [60]. A similar scenario may be evident in the in vivo condition in tumor tissues compared to that of their surrounding tissue. The increased SOD, Cat and GPx activity may be favoured by the elevated NPSH in vivo condition which reduces back the enzymatic catalytic sites. Giving insights that redox modulation by –SH crucially induced modification of antioxidant enzymes activity and favours to maintain a low level of ROS which may activate anti-apoptotic protein Akt [61, 62]. In breast cancer cells, inhibition of the mitochondrial ROS generation suppresses estrogen induced cell proliferation, proposing a role of estrogen mediated mitochondrial ROS in tumor growth [63]. After malignant transformation many cancer cells show a sustained increase in intrinsic reactive oxygen species which maintains the oncogenic phenotype and drives tumor progression. Conclusively, redox adaption through up regulation of anti-apoptotic and antioxidant molecules allows cancer cells to promote survival and to develop resistance to anticancer drugs. Little is known how an increase in intracellular oxidative stress levels is sensed and transduced into ROS-induced specific intracellular signalling to regulate the expression of antioxidant and survival genes [64].

In spite of the extensive biochemical characterization and functional studies of SULT1E1 until recently, little is known about the transcriptional regulation of SULT1E1 under oxidative stress. In our study, SULT1E1 is contrarily expressed in breast cancer patients along with an increased oxidative stress in both the surrounding and tumor. A study reports that SULT1E1 is the transcriptional target of nuclear receptor factor 2 (Nrf2) [65]. Nrf2 remains low under normal conditions but it is induced many-fold in response to endogenous or exogenous stresses or toxicants. Nrf2 target genes function in elimination of reactive oxygen species (ROS) and diminishing inflammation, drug and carcinogen detoxication, and intermediary metabolism [66, 67]. According to the immunohistochemistry results Nrf2 was significantly more in the tumor tissue as compared to the corresponding surrounding (Fig. 8). It indicates that Nrf2 may be the transcriptional regulator of SULT1E1 in case of breast cancers. Our correlation study strongly supports this finding (Table 2). Keap1 negatively regulates Nrf2 by targeting it for subsequent degradation. A study presents evidence that Dipeptide-peptidase 3 (DPP3) binding sequesters KEAP1 in an oxidative stress-inducible manner and enhances the Nrf2 function. Elevated levels of DPP3 mRNA correlate with increased Nrf2 downstream gene expression [68].

Nrf2 has emerged as a key modifier in cancer development, acting in both tumor suppression and tumor promotion functions, depending on context. High levels of Nrf2 in tumors are generally correlated with poor prognosis [69, 70]. SULT1E1 is expressed under oxidative stress as evident from our MDA results (Fig. 1), where Nrf2 is activated (Tables 2 and 3). Eventually, that favours Nrf-2 function which may induce SULT1E1. Another way is that oxidative stress blocks the E3 ligase activity of Keap1 which stabilizes Nrf-2 allowing it to drive the expression of certain antioxidant and drug metabolizing enzyme as noticed in the current study [70]. Thus, oxidative stress favours the Nrf2 pathway of SULT1E1 up-regulation (Table 1).

Breast tumors NPSH level (Fig. 1) may provide a localized reducing environment where active Keap1 may negatively regulate Nrf2 and hinders the SULT1E1 expression as in few patients (Fig. 3). On the other hand dexamethasone (DEX) induced the expression and activity of SULT1E1 through glucocorticoid receptor (GR) [71]. Functional activity of the GR is suppressed under oxidative conditions and restored in the presence of reducing reagents [72]. The GR pathway of SULT1E1 induction is suppressed under oxidative stress. It is likely to be a state where GR pathway and Nrf-2 (if keap1 is not mutated in breast cancer) both remain suppressed under oxidative stress, as may be the case of low SULT1E1 expression in a few breast tumors (Fig. 3). It may be proposed that expression/function of oxidative stress regulated SULT1E1 cannot rely on unidirectional pathways, rather complex and pivotal switching machinery regulates its action.

In an in vitro experiment we found that dexamethasone, a glucocorticoid receptor (GR) activator causes induction of SULT1E1 which helps antagonizing E2 [71]. In an in vivo xenografted-E2 model SULT1E1 expression was significantly reduced compared to control. This indicates that excess E2 regulates its availability via SULT1E1 inhibition. Thus antagonising E2 may not allow E2 to impose its regulatory effect on SULT1E1 expression. Activation of GR induces SULT1E1 which may be an essential pathway in breast cancer patients where GR is influenced by oxidative stress.

To strongly confirm whether Nrf2 is responsible for SULT1E1 expression we also did an in vitro experiment with lansoprazole, a known Nrf2 inducer. Lansoprazole a potent gastric ulcer drug which inhibits proton pump induces anti-oxidative stress via induction of Nrf2 [73]. The cells incubated with lansoprazole showed an elevated SULT1E1. Thus, confirming the fact that Nrf2 causes induction of SULT1E1. This study evidently proofs that the variation in Nrf2 expression and activation as a transcription factor may play an important role in breast cancer patients via induction of SULT1E1 what we have noticed in our immunohistochemical study.

A report showed that aryl hydrocarbon receptor (AhR) knockdown significantly increased SULT1E1 expression. When the breast cells switch to a proliferative state, a lessening of cell–cell contact causes activation of AhR activity and suppression of SULT1E1 expression in tumor as found in our studies (Fig. 3), resulting in increased active estrogen levels in the breast microenvironment [74], interestingly it was found that arsenic-induced AhR activation and -enhanced CYP1A1 expression can be further increased by a pro-oxidant, buthionine-(*S*,*R*)-sulfoximine, and suppressed by antioxidants, such as *N*-acetylcysteine and catalase [75] leading to the conclusion that AhR is active under oxidative stress which may suppress SULT1E1 expression.

To support the above interpretations, we treated female rats only with E2 and found that SULT1E1 expression was decreased both at mRNA and protein level as compared to the control (Figs. 4, 5). At the same time induction of NPSH and reduction of MDA (oxidative stress marker) were noticed. Proposing that local redox environment may regulate SULT1E1 expression. Perhaps tumors expressed SULT1E1 mRNA more than the surrounding (Fig. 5), and SULT1E1 protein was high in few tumors and low in some as compared to that of the surrounding (Fig. 3). The average E2 level was high in tumors along with high MDA. Thus, two different pathways are likely to be controlling the SULT1E1 expression, one via E2 and the other via oxidative stress and both resulted in the carcinogenic transformation of the tissues. A delicate intracellular interplay between oxidizing and reducing equivalents allows ROS and E2 to function as second messengers in the control of cell proliferation and transformation. Estrogen via ER induces transcriptional activation of E2F1 which results in the tamoxifen resistance in breast cancer cells. ER status is not the major determinant of breast cancer progression via estrogen. A summated effect of estrogen and oxidative stress is responsible for breast carcinogenesis where BRCA1/2 deficiency augments sensitivity of breast tissue to both estrogen and oxidative stress [75, 76]. BRCA1 deficiency causes cells to start gaining stem cell feature. BRCA1 mutation coupled with ROS related damages increases the opportunity of oncogenic transformation.

ROS activates NFκβ through IKK degradation [77]. NFκβ is required for normal lobulo-alveolar development of mammary gland [78]. The over-expression or aberrant NFκβ subunits eventually results in the enhanced expression of NFκβ responsive genes like cyclin E that contributes to breast cancer progression, cyclin E is expressed in many breast cancer cell lines and associated with poor prognosis [79]. NFκβ regulates breast cancer metastasis, through up-regulating genes including NOS, COX-2 and VEGF [80–82]. We have noticed a tremendous expression of NFκβ in breast tumor as compared to the surrounding (Fig. 8) which supports that NFκβ is one important factor that is associated with breast cancer progression (Table 2). Modifications in the cellular thiol redox state, due to Nrf2 induction of antioxidants expression, may affect the phosphorylation of critical residues of NFκβ that contribute to its nuclear import [83]. In the current correlation study level of significance found to p = 0.075, and due to the inter-individual variability correlation was not significant. But the relation was noticed to be positive (r = 0.993) (Table 2). Activation of NFκβ is inhibited by phospholipid hydroperoxide glutathione peroxidase and 15-lipoxygenase accompanying up-regulation of HO-1, via Nrf2 activation [84]. We have noticed a significant elevated GPx activity in tumor (Fig. 2 GPx), Intending NFκβ inhibition and induction of apoptosis. Interestingly NFκβ-inhibited acute myeloid leukaemia cells do not undergo TNF-induced apoptosis and hemeoxygense-1 (HO-1) is found to resist apoptosis [85].

The anti-apoptotic effect of HO-1 induced by Nrf2 may be mediated via carbon monoxide [86]. Therefore our study suggests that NFκβ remains-moderately inhibited via GPx, overexpression and activation of Nrf2 expresses HO-1 which inhibits apoptosis to an extent leading to disease severity. Findings strongly support the participation of NFκβ p65 in the negative regulation of Nrf2 signalling via depriving CBP (CREB binding protein) from Nrf2 or recruitment of Histone deacetylase 3 (HDAC3) on antioxidant recruitment element (ARE) or Mafk providing a new insight into a possible role of NFκβ in suppressing the expression of anti-inflammatory or anti-tumor genes [87]. Hence our study shows that severe stages of breast cancer maintains a perfect balance of oxidants, antioxidant, E2, SULT1E1 along with genes responsible for proliferation and apoptosis via Nrf2 and NFκβ (Table 2). Tumor cells/cancer stem cells are dependent on their antioxidant capacity and may become vulnerable to agents that diminish antioxidant systems.

The accumulation of p62 (a substrate of autophagy) being the link between Nrf-2 and NF-κB expressions is of great importance in death/survival of tumor cells. Nrf-2 dependant autophagy via the substrate p62 accumulation leads to (a) Nrf-2 stabilization and (b) NF-κB activation. The Nrf-2 stabilization by p62 imparts tumor cells with resistance to hypoxic stress. Moreover, retention of damaged organelles, including mitochondria support tumor cells. For example, temozolomide (TMZ), an alkylating agent used to treat glioblastoma multiforme (GBM) and anaplastic astrocytoma, induces autophagy and subsequent therapeutic resistance, which is why Nrf2 inhibitors exhibit a therapeutic effect when used in combination with (TMZ) [88]. Autophagy in cancer cells may have bimodal response of pro-death or a pro-survival role. Lower rate of autophagy may result in catabolic degradation of nonessential cellular proteins and debris creating a large amino acid pool that eventually helps in cancer cell survival. In contrary, uncontrolled autophagy mimicking to apoptotic cell death results in large scale tumor cell death [88, 89]. Hence, autophagy can either promote or suppress the survival and proliferation in the tumor microenvironment. The proinflammatory induction of transcriptional regulation by the NF-κB has a great influence on tumor cell survival.

This has recently been explained in human breast cancer and earlier demonstrated in other, like bladder cancers that zinc finger E-box binding homeobox 1 (ZEB1) is associated with the development of epithelial to mesenchymal transition (EMT). The EMT has been shown to be the stepping stone in different types of cancers including breast cancer [90]. A transcription regulator ZEB1 targets E-cadherin repression which is a prerequisite for EMT state. So, it is noteworthy that the EMT status linked to ZEB1 expression does not only indicate the mechanistic steps of disease pathogenesis but also it could be a potential therapeutic target [90, 91]. Moreover, ZEB1 protein expression has some predictive outcome during neoadjuvant therapy in breast cancer patients. Intra-tumoral expression of ZEB1 is of great importance because it would demonstrate the EMT pattern in the diseased tissues with comparison to the control surrounding tissues. In this regard, further studies are necessary.

The strategy of the therapeutic approach in breast cancer depends on the stages of the disease. Recent approaches includes from combined chemotherapeutic to radio-therapeutic measures. Doxorubicin (DOX) is one of the preferred drugs for treating breast and liver cancers. Recent study shows that the efficacy of DOX is significantly increased by cholesterol depleting agent methyl-β-cyclodextrin (MCD) with an involvement of p53. The p53 activation has been shown to be mediated by the induction of FasR/FasL (Fas Receptor Ligand) pathway [92]. Breast cancers with positive expression of Estrogen Receptor (ER+) are treated with anti-hormone/endocrine therapy which targets the activity of the receptor. Ability of genomics in unraveling rare mutations and gene rearrangements that may impact the development of resistance and therefore treatment of ER+ breast cancer [93]. Cannabinoids (CBs) from *Cannabis sativa* CBs are already administered to breast cancer patients at advanced stages of the disease, but they might also be effective at earlier stages to decelerate tumor progression. In human epidermal growth factor receptor 2-positive and triple-negative breast cancer cells, blocking protein kinase B- and cyclooxygenase-2 signaling via CB2-R prevents tumor progression and metastasis [94]. Proper staging is critical for determining the appropriate clinical treatment course and surgical planning. Neoadjuvant therapies, where patients receive systemic therapy before surgical removal of the tumor, can downstage tumors allowing breast-conserving surgery, rather than mastectomy [91, 95]. The effectiveness of standard and/or potential new therapies can be tested in the neoadjuvant pre-surgical setting [91]. It can potentially help to identify markers differentiating patients that will potentially benefit from continuing with the same or a different adjuvant treatment enabling personalised treatment [95].

## Conclusions

In the current study it is clearly demonstrated that intracellular redox state may influence the E2 metabolizing enzyme like SULT1E1 expressions and the functions of several antioxidant enzymes. Moreover redox-regulated Nrf-2 and NFκβ has a strong correlation with SULT1E1 expression and the disease severity. The human sample number was rather less due to several regulatory affairs and consent of the patients. And also, due to the stringent exclusion criteria a large number of patients having several other diseases/radiation therapies or frequent medications were excluded. We were very much concerned about the fact; so, we conducted the experiments extensively at cellular, biochemical and molecular level. We evaluated protein expression by WB and verified its localization by IHC. Several gene expression studies were done. Large number of experiments were tested both in in vivo and in vitro model. DNA stability and tissue architecture were verified. Moreover, rat model xenografted with human breast cancer cells were tested. An extensive statistical analysis was made to validate our findings at biochemical and molecular levels.

Some prospective treatments may aim to intensely increase intracellular ROS by decreasing antioxidant capacity in cancer cell to kill them. A cancer cell utilizes the antioxidant pool to maintain the ROS just above the level required to initiate cancer and below the toxic threshold that may kill them. It becomes evident that a much more detailed understanding of ROS and E2-mediated signalling in tumor cells is necessary to develop new strategies. Based on redox modification of different important protein in the current study suitable therapeutic strategies may be adopted to specifically kill cancer cells.

## Data Availability

The datasets used and/or analysed during the current study available from the corresponding author on request.

## Abbreviations

Cat: Catalase
DAB: Diaminobenzidine
DTNB 5,5′: Dithiobis-2-nitrobenzoic acid
ER: Estrogen-receptor
GPx: Glutathione peroxidase
HCV: Hepatitis C virus
HIV: Human immunodeficiency viruses
HPV: Human papillomavirus
IDC: Invasive ductal carcinoma
IHC: Immunohistochemistry
MDA: Malondialdehyde
NBT: Nitro blue tetrazolium
NFκβ: Nuclear factor kappa-light-chain-enhancer of activated B cells
NPSH: Non-protein-soluble-thiol
Nrf2: Nuclear-receptor-factor 2
SOD: Superoxide dismutase
SULT1E1: Estrogen sulfotransferase
TEMED: Tetra-methylethylene diamine
